# Supplementary material for: Heterogenous humoral and cellular immune responses with distinct trajectories post-SARS-CoV-2 infection in a population-based cohort
Source: Nat Commun. 2022 Aug 18;13:4855. doi: 10.1038/s41467-022-32573-w (PMC9386650; doi:10.1038/s41467-022-32573-w)
Supplement: Supplementary file 1 — Supplementary Information [file 41467_2022_32573_MOESM1_ESM.pdf]

Supplementary Information

Heterogenous Humoral and Cellular Immune Responses with Distinct Trajectories Post-SARS-CoV-2 Infection in a Population-Based Cohort

Dominik Menges[1]†, Kyra D. Zens[1,2]†, Tala Ballouz[1]†, Nicole Caduff[1,2], Daniel Llanas-Cornejo[1], Hélène E. Aschmann[1,3], Anja Domenghino[1,4], Céline Pellaton[5], Matthieu Perreau[5], Craig Fenwick[5], Giuseppe Pantaleo[5], Christian R. Kahlert[6,7], Christian Münz[2], Milo A. Puhan[1]*, Jan S. Fehr[1]

Affiliations:

[1] Epidemiology, Biostatistics and Prevention Institute (EBPI), University of Zurich (UZH), Zurich, Switzerland.

[2] Institute for Experimental Immunology, University of Zurich (UZH), Zurich, Switzerland.

[3] Department of Epidemiology and Biostatistics, University of California San Francisco, San Francisco, USA.

[4] Department of Visceral and Transplantation Surgery, University Hospital Zurich (USZ), University of Zurich (UZH), Zurich, Switzerland.

[5] Service of Immunology and Allergy, Lausanne University Hospital (CHUV), University of Lausanne (UNIL), Lausanne, Switzerland

[6] Division of Infectious Diseases and Hospital Epidemiology, Cantonal Hospital St. Gallen, St. Gallen, Switzerland

[7] Division of Infectious Diseases and Hospital Epidemiology, Children's Hospital of Eastern Switzerland, St. Gallen, Switzerland

* Corresponding author. Email: miloalan.puhan@uzh.ch.

† These authors contributed equally to this work.

Supplementary Figures

Supplementary Figure 1. Antibody positivity in overall population and subsample over time and validation of Luminex assay.

Supplementary Figure 2. Neutralizing antibody and T cell positivity over time and estimation of T cell decay kinetics.

Supplementary Figure 3. Concordance of antibody and T cell positivity or negativity over time.

Supplementary Figure 4. Antibody, neutralizing antibody, and T cell responses, and cellular subsets within clusters.

Supplementary Figure 5. Study participant flow chart.

Supplementary Figure 6. Gating strategy for flow cytometric analyses.

Supplementary Tables

Supplementary Table 1. Study population characteristics.

Supplementary Table 2. Anti-S IgA and IgG antibody responses in the overall study population over time.

Supplementary Table 3. Antibody, neutralizing antibody, and T cell responses in the subsample over time.

Supplementary Table 4. Sensitivity analysis regarding anti-S IgA and IgG antibody responses, weighted by age group.

Supplementary Table 5. Detailed results for antibody, neutralizing antibody, and T cell decay estimation.

Supplementary Table 6. Association between demographic and clinical factors and anti-S IgG antibody responses over time.

Supplementary Table 7. Association between demographic and clinical factors and anti-S IgA antibody responses over time.

Supplementary Table 8. Association between demographic and clinical factors and pooled T cell responses over time.

Supplementary Table 9. Association between demographic and clinical factors and anti-S IgG antibody positivity at two weeks and six months.

Supplementary Table 10. Association between demographic and clinical factors and overall T cell positivity at two weeks and six months.

Supplementary Table 11. Reference table for converting anti-S IgG MFI ratios to U/ml.

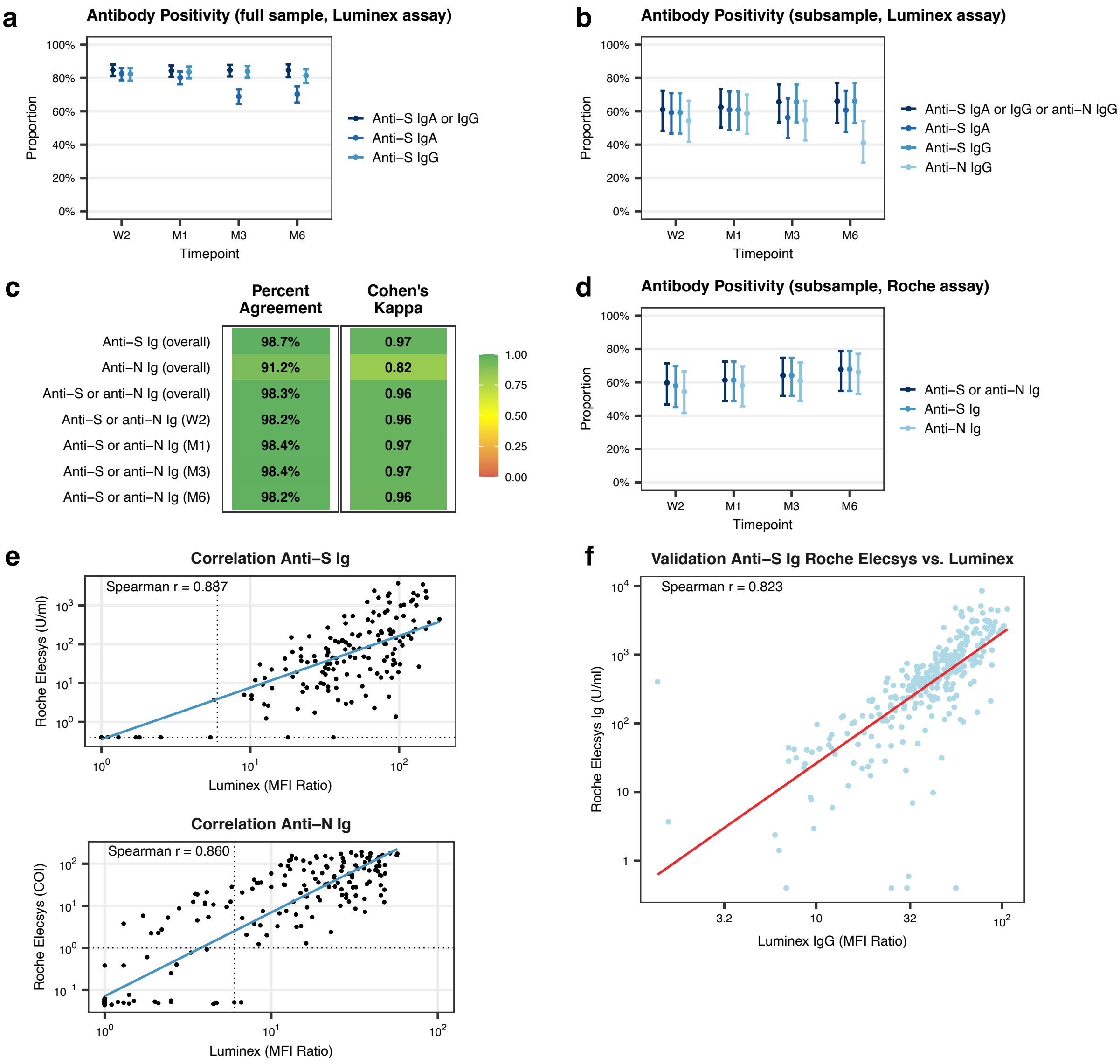

Supplementary Figure 1. **Antibody positivity in overall population and subsample over time and validation of Luminex assay.** (**a**) Seropositivity for anti-S IgA and anti-S IgG antibodies over time in the overall study population (total n=431) based on Luminex assay mean fluorescence intensity (MFI) ratios (using MFI ratio cutoff values of 6.5 for IgA and 6.0 for IgG). Points and error bars in panels a–b and d represent estimated proportions with associated 95% Wilson confidence intervals. W2: two weeks, M1: one month, M3: three months, M6: six months after diagnosis. (**b**) Seropositivity for anti-S IgA, anti-S IgG, and anti-N IgG antibodies over time as in panel a, for the subsample selected for detailed analyses (total n=64). (**c**) Validation of antibody test results comparing Luminex and Roche Elecsys assays. For each individual, results were categorized as positive (detectable response) or

negative (no detectable response) based on cutoff values of an MFI ratio of 6.0 for Luminex anti-S and anti-N IgG assays, a concentration of >0.8U/ml for the Roche Elecsys anti-S Ig assay, or a cutoff index (COI) value of 1.0 for the Roche Elecsys N Ig assay. Percent agreement and Cohen's Kappa for anti-S Ig, anti-N Ig, and combined anti-S Ig or anti-N Ig between assays are shown. (**d**) Seropositivity for anti-S Ig and anti-N Ig antibodies over time in the subsample based on Roche Elecsys assays based on cutoff values of a concentration of >0.8U/ml for S Ig and a COI value of 1.0 for N Ig. (**e**) Correlation of anti-S Ig and anti-N Ig antibody levels based on Luminex (MFI ratio) and Roche Elecsys (U/ml or COI) assays. Blue lines represent regression lines for respective comparisons based on unadjusted linear regression models. (**f**) Correlation of anti-S Ig results based on Luminex (MFI ratio) and Roche Elecsys (U/ml) assays based on external cross-validations using samples of SARS-CoV-2-infected individuals of the Lausanne University Hospital, Switzerland (n=298; individuals were not part of the reported population-based study). Red line represents regression equation estimated for conversion of Luminex anti-S IgG MFI ratios to Roche Elecsys anti-S Ig concentration in U/ml. Source data are provided as a Source Data file.

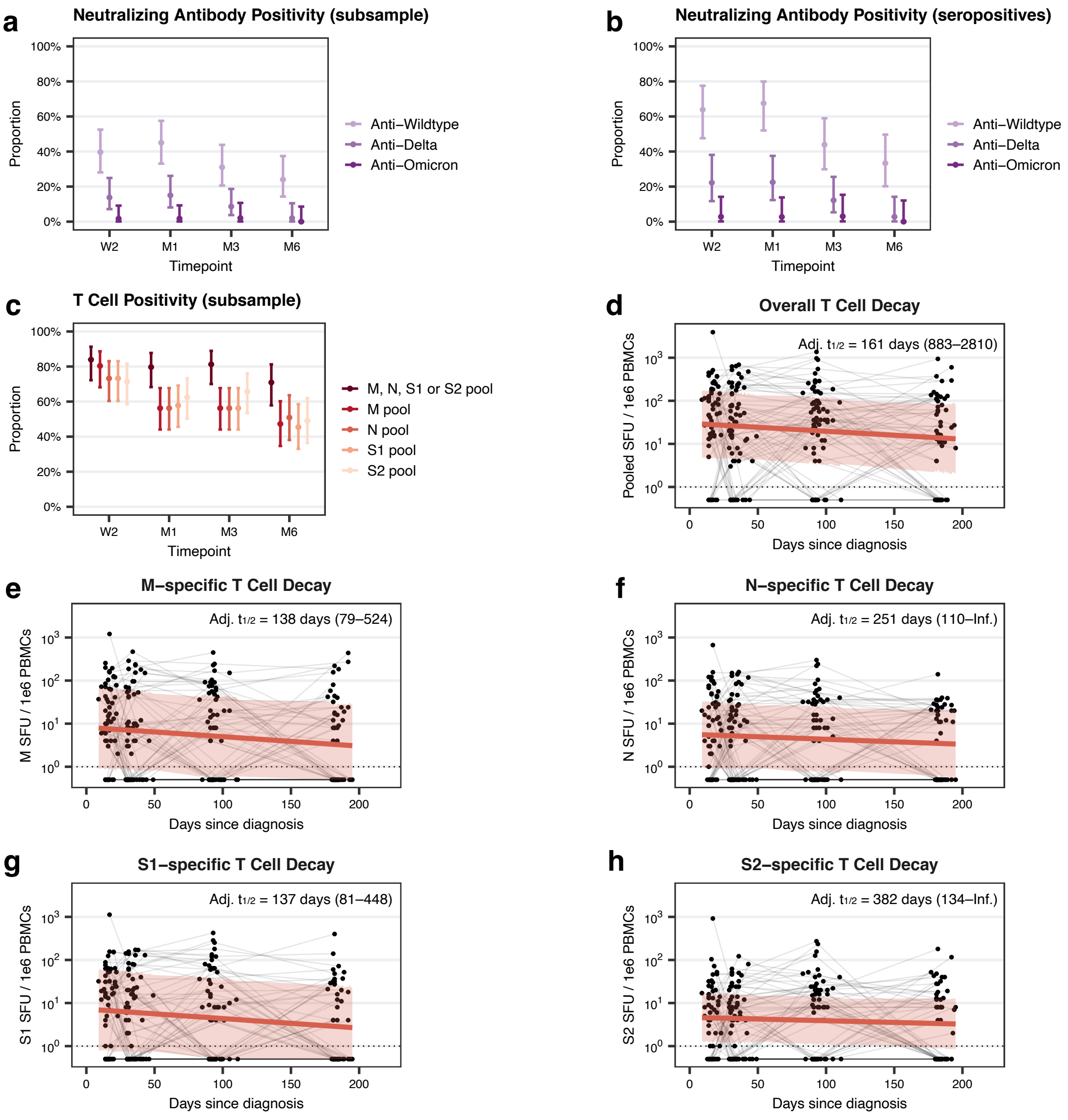

Supplementary Figure 2. Neutralizing antibody and T cell positivity over time and estimation of T cell decay kinetics. (**a**) Positivity for anti-Wildtype, anti-Delta and anti-Omicron neutralizing antibodies over time in subsample (total n=64). Results were categorized as positive (detectable response) or negative (no detectable response) based on an IC_{50} cutoff value of 50 or higher. Points and error bars in panels a–c represent estimated proportions with associated 95% Wilson confidence intervals. W2: two weeks, M1: one month, M3: three months, M6: six months after diagnosis. (**b**) Positivity for neutralizing antibodies over time among subsample participants that were tested seropositive for anti-S IgA, anti-S IgG, or anti-N IgG. (**c**) Positivity for SARS-CoV-2-specific T cells over time among individuals in the subsample as assessed by ELISpot. Positive (detectable) T cell responses to

individual peptide pools (M, N, S1, or S2) were defined as a spot-forming unit (SFU) value of greater than 0. For overall T cell responses, individuals were considered positive if they were positive to one or more peptide pools. (**d**) Decay estimation for pooled T cell responses (summed M, N, S1, and S2 pool-specific SFU per 1e6 peripheral blood mononuclear cells (PBMCs)) based on mixed linear regression model. Lines and error bands in panels d–h represent regression lines with associated 95% confidence intervals estimated using bootstrap. Dotted lines in panels d–h represent limit of detection cutoff (SFU values greater than 0). Adj. $t_{1/2}$: half-life based on model adjusted for time since diagnosis, age group, sex and symptom count, using a random intercept for each individual in the study, Inf.: Infinity. (**e**) Decay estimation for M-specific T cells based on mixed linear regression model (adjustment as in panel d). (**f**) Decay estimation for N-specific T cells based on mixed linear regression model (adjustment as in panel d). (**g**) Decay estimation for S1-specific T cells based on mixed linear regression model (adjustment as in panel d). (**h**) Decay estimation for S2-specific T cells based on mixed linear regression model (adjustment as in panel d). Source data are provided as a Source Data file.

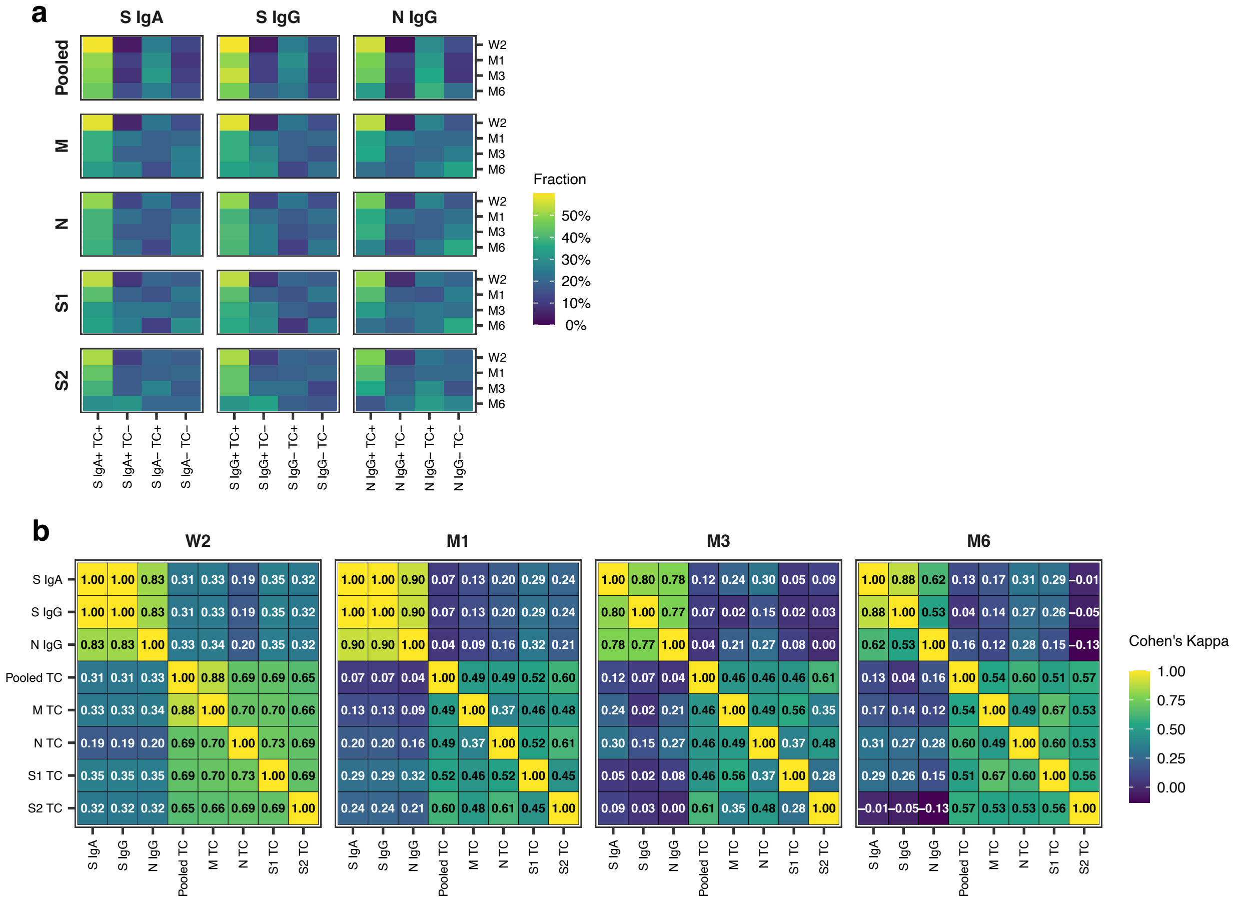

Supplementary Figure 3. Concordance of antibody and T cell positivity or negativity over time. (**a**) Within the subsample (total n=64), proportion of participants with concordant results between being positive (detectable response) or negative (no detectable response) for anti-S IgA, anti-S IgG, and anti-N IgG antibody subtypes (MFI ratio value above the limits of detection of 6.5 for IgA and 6.0 for IgG) and between being positive or negative for overall T cell responses (detectable SFU to at least one peptide pool), or to individual M, N, S1 or S2 peptide pools (SFU value of greater than 0 within each peptide pool) over time. W2: two weeks, M1: one month, M3: three months, M6: six months after diagnosis. (**b**) Concordance as in panel a calculated based on Cohen's Kappa. Numbers in individual cells correspond to respective Cohen's Kappa values. Source data are provided as a Source Data file.

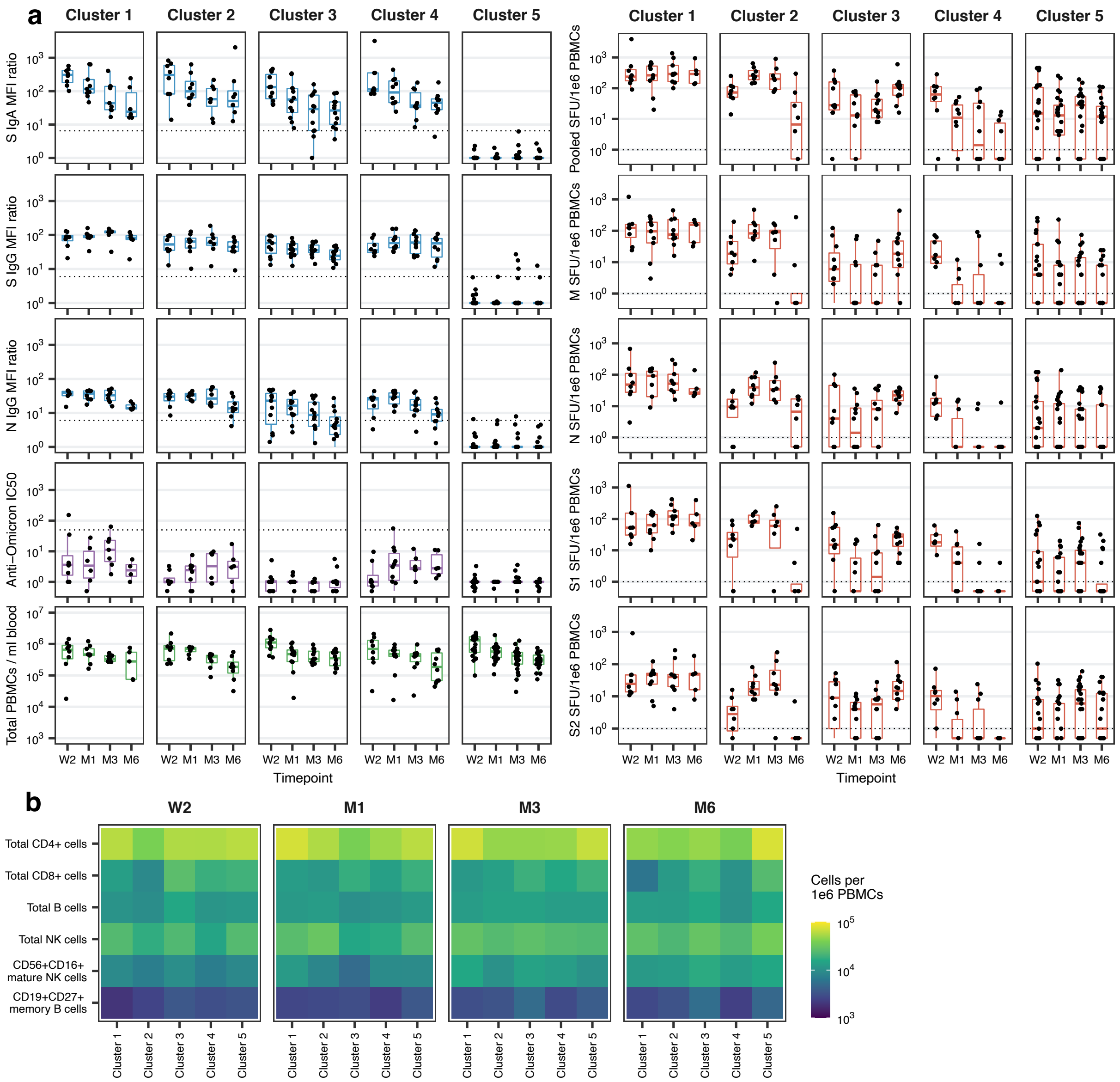

Supplementary Figure 4. **Antibody, neutralizing antibody, and T cell responses, and cellular subsets within clusters.** (**a**) Results from antibody testing, neutralizing antibody testing, ELISpot and flow cytometric analyses in the detailed subsample (n=64), stratified by cluster. Plots demonstrate results for anti-S IgA, anti-S IgG, and anti-N IgG mean fluorescence intensity (MFI) ratios, anti-Omicron neutralizing antibody half maximal inhibitory concentration (IC_{50}), total peripheral blood monocytic cells (PBMCs) per ml blood, pooled T cell responses (summed M, N, S1, and S2 spot-forming units (SFU) per 1e6 PBMCs), and M, N, S1, and S2 epitope pool-specific T cells (SFU per 1e6 PBMCs). Boxplots represent median and interquartile range (IQR; whiskers: 1.5*IQR) at

respective timepoints. Dotted lines indicate limit of detection cutoffs (6.5 for IgA MFI ratios, 6.0 for IgG MFI ratios, 50 for half maximal inhibitory concentrations (IC_{50}) for neutralizing activity, and SFU values greater than 0 for T cell responses). W2: two weeks, M1: one month, M3: three months, M6: six months after diagnosis. (**b**) Results from flow cytometric analyses stratified by cluster. Heatmap demonstrates the total number of $CD4^+$ T cells, $CD8^+$ T cells, B cells, Natural Killer (NK) cells, $CD56^+CD16^+$ mature NK cells, and $CD19^+CD27^+$ memory B cells per 1e6 PBMCs. Source data are provided as a Source Data file.

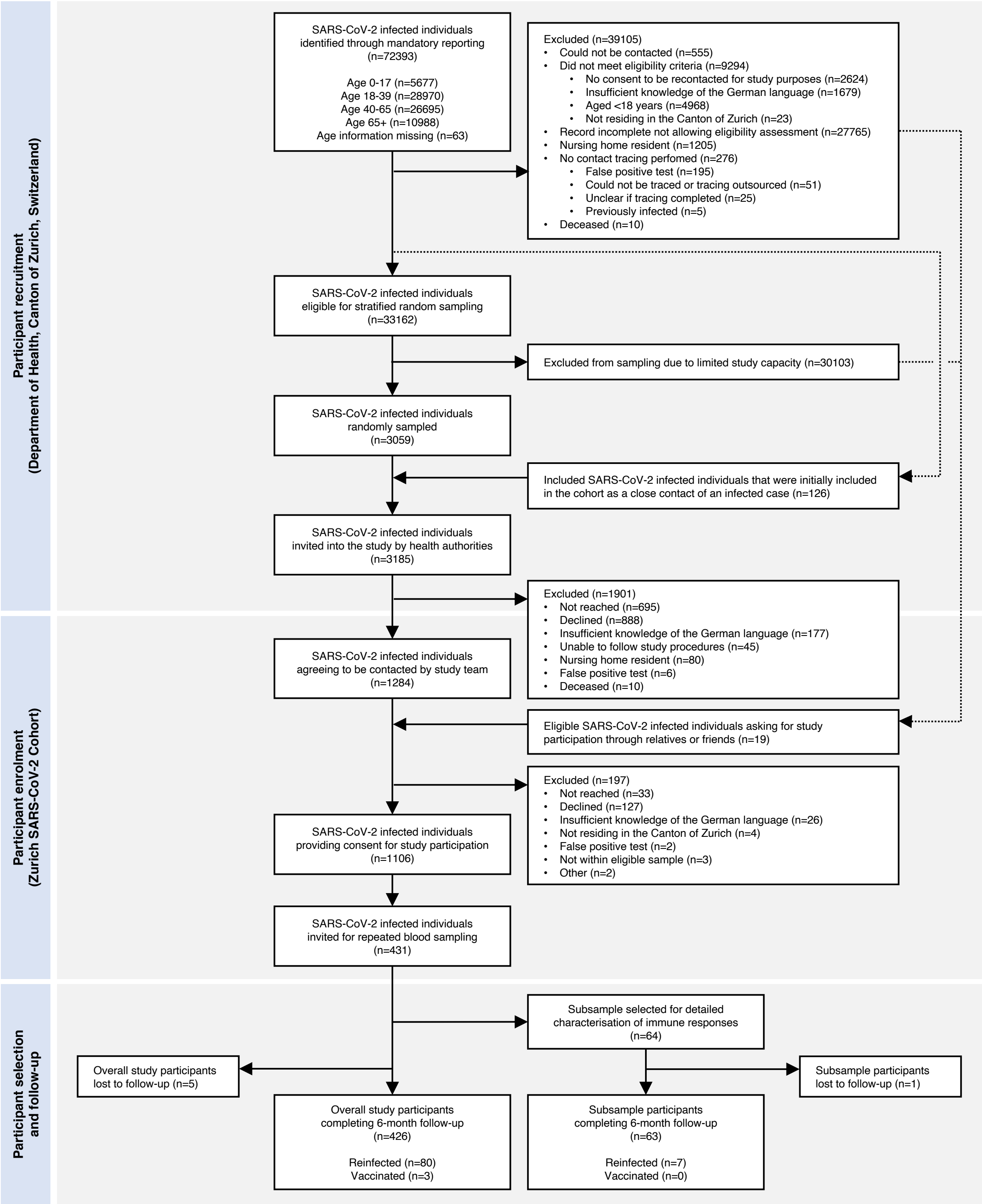

Supplementary Figure 5. Study participant flow chart. Figure depicting the enrolment, selection and follow-up of study participants.

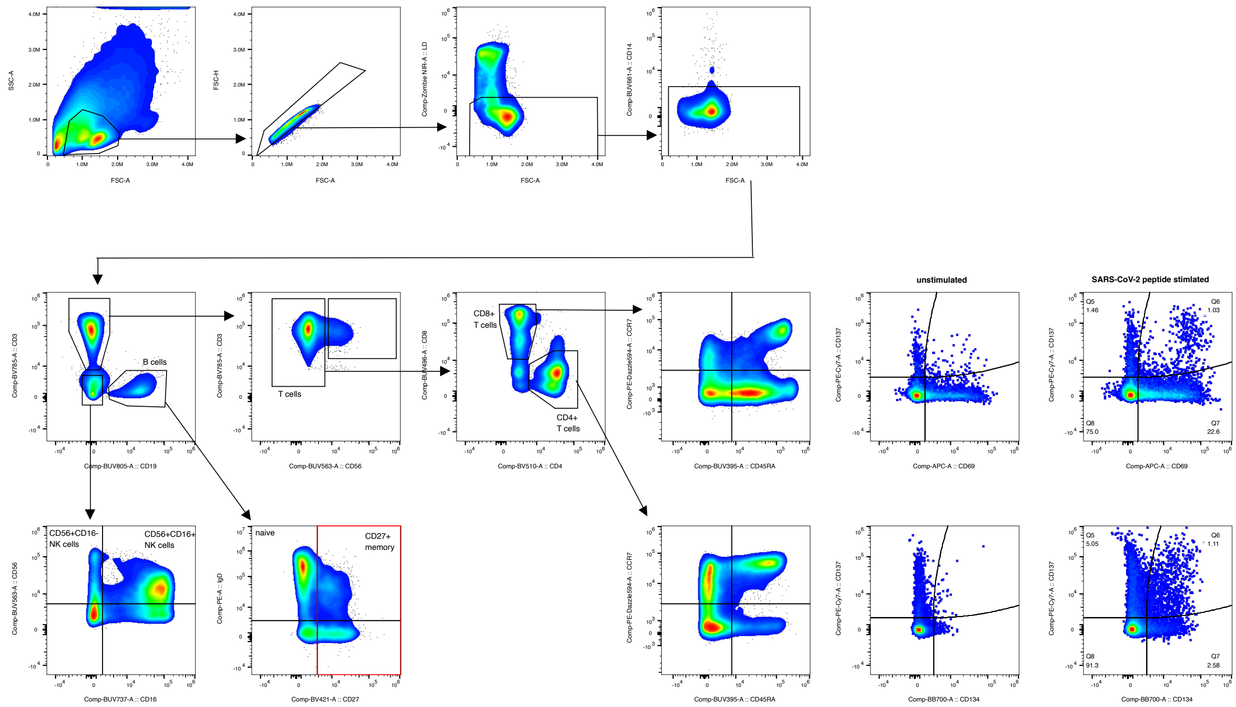

Supplementary Figure 6. Gating strategy for flow cytometric analyses. Figure depicting the gating strategy applied for flow cytometric analyses.

Supplementary Table 1. **Study population characteristics.** Characteristics of the overall study sample (n=431) and of the subsample selected for detailed analyses (n=64). Study participants were recruited between the 06th of August 2020 and the 26th of January 2021. IQR: Interquartile Range, SD: Standard Deviation. Source data are provided as a Source Data file.

	**Overall Study Population (N=431)**	**Subsample Participants (N=64)**	**Participants not in the Subsample (N=367)**
**Age**			
Mean (SD)	51.7 (18.3)	52.1 (18.9)	51.6 (18.3)
Median (IQR)	52.0 (35.0 to 68.0)	53.5 (33.5 to 68.0)	52.0 (35.0 to 68.0)
Range	18 to 88	18 to 87	18 to 88
**Age group**			
18-39 years	135 (31.3%)	21 (32.8%)	114 (31.1%)
40-64 years	144 (33.4%)	21 (32.8%)	123 (33.5%)
65+ years	152 (35.3%)	22 (34.4%)	130 (35.4%)
**Sex**			
female	212 (49.2%)	36 (56.2%)	176 (48.0%)
male	219 (50.8%)	28 (43.8%)	191 (52.0%)
**Symptom count at diagnosis**			
Asymptomatic	76 (17.6%)	20 (31.2%)	56 (15.3%)
1-5 symptoms	163 (37.8%)	17 (26.6%)	146 (39.8%)
≥6 symptoms	192 (44.5%)	27 (42.2%)	165 (45.0%)
**Symptom count at diagnosis**			
Mean (SD)	5.1 (3.8)	5.2 (5.4)	5.1 (3.5)
Median (IQR)	5.0 (2.0 to 7.0)	4.0 (0.0 to 8.5)	5.0 (3.0 to 7.0)
Range	0 to 20	0 to 20	0 to 16
**Self-reported symptom severity at diagnosis**			
None	74 (17.3%)	20 (31.7%)	54 (14.8%)
Mild to moderate	281 (65.7%)	30 (47.6%)	251 (68.8%)
Severe to very severe	73 (17.1%)	13 (20.6%)	60 (16.4%)
Missing	3 (0.7%)	1 (1.6%)	2 (0.5%)
**Hospitalization within first 2 weeks**			
No	413 (95.8%)	53 (82.8%)	360 (98.1%)
Yes	18 (4.2%)	11 (17.2%)	7 (1.9%)
**ICU admission within first 2 weeks**			
No	429 (99.5%)	63 (98.4%)	366 (99.7%)
Yes	2 (0.5%)	1 (1.6%)	1 (0.3%)
**Vaccinated at 6 months follow-up**			
No	351 (81.4%)	57 (89.1%)	294 (80.1%)
Yes	80 (18.6%)	7 (10.9%)	73 (19.9%)

**Reinfected at 6 months follow-up**			
No	428 (99.3%)	64 (100.0%)	364 (99.2%)
Yes	3 (0.7%)	0 (0.0%)	3 (0.8%)
**Smoking status**			
Non-smoker	261 (61.3%)	29 (46.0%)	232 (63.9%)
Ex-smoker	106 (24.9%)	20 (31.7%)	86 (23.7%)
Smoker	59 (13.8%)	14 (22.2%)	45 (12.4%)
Missing	5 (1.2%)	1 (1.6%)	4 (1.1%)
**Body mass index**			
Mean (SD)	24.5 (4.1)	25.4 (4.5)	24.3 (4.0)
Missing	5 (1.2%)	1 (1.6%)	4 (1.1%)
**Comorbidities**			
No	300 (69.6%)	44 (68.8%)	256 (69.8%)
Yes	131 (30.4%)	20 (31.2%)	111 (30.2%)
**Immune suppression**			
No	410 (97.4%)	61 (98.4%)	349 (97.2%)
Yes	11 (2.6%)	1 (1.6%)	10 (2.8%)
Missing	10 (2.3%)	2 (3.1%)	8 (2.2%)
**Employment**			
Employed	258 (59.9%)	37 (57.8%)	221 (60.2%)
Student	22 (5.1%)	4 (6.2%)	18 (4.9%)
Retired	124 (28.8%)	18 (28.1%)	106 (28.9%)
Unemployed or other	27 (6.3%)	5 (7.8%)	22 (6.0%)
**Education**			
None or mandatory school	18 (4.2%)	4 (6.2%)	14 (3.8%)
Vocational training or specialized baccalaureate	179 (41.5%)	27 (42.2%)	152 (41.4%)
Higher technical school or college	113 (26.2%)	14 (21.9%)	99 (27.0%)
University	121 (28.1%)	19 (29.7%)	102 (27.8%)
**Nationality**			
Swiss	376 (87.2%)	53 (82.8%)	323 (88.0%)
Non-Swiss	55 (12.8%)	11 (17.2%)	44 (12.0%)

Supplementary Table 2. **Anti-S IgA and IgG antibody responses in the overall study population over time.** Detailed test results for anti-S IgA, anti-S IgG, or combined anti-S antibody responses in the full study population (total n=431) at two weeks, one month, three months and six months after diagnosis of SARS-CoV-2 infection. Antibody responses were measured as mean fluorescence intensity (MFI) ratios compared to pre-pandemic seronegative control samples using a Luminex-based assay. For each individual, results were positive (detectable response) or negative (no detectable response) based on MFI ratio cutoff values of 6.5 for IgA and 6.0 for IgG. Data measured after vaccination or known reinfection were omitted for immune trajectory analyses. As a reference for other studies, anti-S IgG MFI ratios were converted into U/ml using a formula derived from cross-validation studies using Roche Elecsys Anti-SARS-CoV-2 Ig testing (Supplementary Fig. 1f). IQR: Interquartile Range, SD: Standard Deviation. Source data are provided as a Source Data file.

	**Overall Study Population**			
	**2 Weeks (N=403)**	**1 Month (N=421)**	**3 Months (N=418)**	**6 Months (N=334)**
**Anti-S IgA**				
negative	70 (17.4%)	83 (19.7%)	130 (31.1%)	99 (29.6%)
positive	333 (82.6%)	338 (80.3%)	288 (68.9%)	235 (70.4%)
**Anti-S IgA (MFI ratio)**				
Mean (SD)	137.4 (242.4)	75.1 (132.0)	38.0 (90.1)	39.5 (149.2)
Median (IQR)	65.1 (18.8 to 154.8)	36.0 (9.9 to 77.7)	12.7 (5.1 to 35.8)	12.8 (4.8 to 31.1)
Range	1 to 3216	1 to 1179	1 to 1204	1 to 2058
**Anti-S IgG**				
negative	71 (17.6%)	69 (16.4%)	67 (16.0%)	62 (18.6%)
positive	332 (82.4%)	352 (83.6%)	351 (84.0%)	272 (81.4%)
**Anti-S IgG (MFI ratio)**				
Mean (SD)	38.7 (33.9)	44.0 (37.7)	41.5 (37.5)	27.6 (27.3)
Median (IQR)	32.2 (11.8 to 57.9)	37.1 (16.1 to 61.8)	30.4 (13.6 to 60.5)	21.1 (8.2 to 36.4)
Range	1 to 158	1 to 185	1 to 187	1 to 156
**Anti-S IgG (MFI ratio converted to U/ml for Roche Elecsys anti-S Ig)**				
Mean (SD)	689.2 (1044.4)	873.1 (1352.3)	812.1 (1316.2)	394.7 (791.7)
Median (IQR)	277.0 (41.2 to 875.6)	365.2 (73.1 to 995.9)	248.5 (53.1 to 954.2)	121.8 (21.2 to 351.4)
Range	1 to 6452	1 to 8787	1 to 8978	1 to 6234
**Anti-S IgA or IgG**				
negative	61 (15.1%)	66 (15.7%)	64 (15.3%)	51 (15.3%)
positive	342 (84.9%)	355 (84.3%)	354 (84.7%)	283 (84.7%)

Supplementary Table 3. **Antibody, neutralizing antibody, and T cell responses in subsample over time.** Detailed test results for anti-S IgA or IgG and anti-N IgG antibodies, anti-Wildtype, anti-Delta, and anti-Omicron neutralizing antibodies, as well as M, N, S1, and S2 pool-specific T cell responses in the subsample selected for detailed analyses (total n=64) at two weeks, one month, three months and six months after diagnosis of SARS-CoV-2 infection. Antibody responses were measured using a Luminex-based assay (results expressed as mean fluorescence intensity (MFI) ratios compared to pre-pandemic seronegative control samples) as well as Roche Elecsys anti-S and anti-N Ig assays (results expressed as U/ml or cutoff index (COI), respectively). For each individual, results were considered positive (detectable response) or negative (no detectable response) based on cutoff values of an MFI ratio of 6.5 for IgA and 6.0 for IgG (Luminex), or on cutoff values of >0.8U/ml for anti-S Ig and a COI value of 1.0 for anti-N Ig (Roche Elecsys). Neutralizing antibody responses were quantified as the half maximal inhibitory concentration (IC_{50}), with positive or negative results defined by a cutoff value of 50 or higher. Positive T cell responses to individual peptide pools (M, N, S1, or S2) were defined as a spot-forming unit (SFU) value of greater than 0. For overall T cell responses, individuals were considered positive if they were positive to one or more peptide pools. IQR: Interquartile Range, SD: Standard Deviation. Source data are provided as a Source Data file.

	**Subsample Participants**			
	**2 Weeks (N=59)**	**1 Month (N=64)**	**3 Months (N=64)**	**6 Months (N=56)**
**Luminex-based Assay**				
**Anti-S IgA**				
negative	24 (40.7%)	25 (39.1%)	28 (43.8%)	22 (39.3%)
positive	35 (59.3%)	39 (60.9%)	36 (56.2%)	34 (60.7%)
**Anti-S IgA (MFI ratio)**				
Mean (SD)	201.9 (446.2)	97.5 (155.8)	44.0 (70.0)	70.5 (275.2)
Median (IQR)	82.1 (1.0 to 274.9)	41.0 (1.0 to 120.8)	12.4 (1.0 to 49.8)	16.2 (1.0 to 47.5)
Range	1 to 3216	1 to 646	1 to 407	1 to 2058
**Anti-S IgG**				
negative	24 (40.7%)	25 (39.1%)	22 (34.4%)	19 (33.9%)
positive	35 (59.3%)	39 (60.9%)	42 (65.6%)	37 (66.1%)
**Anti-S IgG (MFI ratio)**				
Mean (SD)	37.2 (39.4)	41.0 (43.6)	45.2 (49.2)	31.6 (33.2)
Median (IQR)	24.0 (1.0 to 73.5)	33.2 (1.0 to 72.6)	29.6 (1.0 to 64.8)	19.2 (1.0 to 47.0)
Range	1 to 129	1 to 159	1 to 187	1 to 120
**Anti-N IgG**				

negative	27 (45.8%)	26 (41.3%)	29 (45.3%)	33 (58.9%)
positive	32 (54.2%)	37 (58.7%)	35 (54.7%)	23 (41.1%)
Missing	*0 (0%)*	*1 (1.6%)*	*0 (0%)*	*0 (0%)*
**Anti-N IgG (MFI ratio)**				
Mean (SD)	16.7 (17.1)	17.3 (16.4)	15.2 (16.7)	8.1 (8.8)
Median (IQR)	8.4 (1.0 to 31.4)	13.6 (1.0 to 31.4)	8.2 (1.0 to 23.8)	4.3 (1.0 to 12.9)
Range	1 to 48	1 to 46	1 to 57	1 to 39
Missing	*0 (0%)*	*1 (1.6%)*	*0 (0%)*	*0 (0%)*
**Anti-S IgA or IgG**				
negative	24 (40.7%)	25 (39.1%)	22 (34.4%)	19 (33.9%)
positive	35 (59.3%)	39 (60.9%)	42 (65.6%)	37 (66.1%)
**Anti-S IgG or anti-N IgG**				
negative	23 (39.0%)	24 (37.5%)	22 (34.4%)	19 (33.9%)
positive	36 (61.0%)	40 (62.5%)	42 (65.6%)	37 (66.1%)
**Anti-S IgA or IgG or anti-N IgG**				
negative	23 (39.0%)	24 (37.5%)	22 (34.4%)	19 (33.9%)
positive	36 (61.0%)	40 (62.5%)	42 (65.6%)	37 (66.1%)
**Roche Elecsys Assay**				
**Anti-S Ig**				
negative	24 (42.1%)	24 (38.7%)	23 (35.9%)	18 (32.1%)
positive	33 (57.9%)	38 (61.3%)	41 (64.1%)	38 (67.9%)
Missing	*2 (3.4%)*	*2 (3.1%)*	*0 (0%)*	*0 (0%)*
**Anti-S Ig (U/ml)**				
Mean (SD)	49.2 (190.4)	128.3 (315.0)	272.6 (636.4)	320.1 (691.4)
Median (IQR)	2.6 (0.4 to 19.2)	12.2 (0.4 to 99.8)	28.9 (0.4 to 165.8)	33.7 (0.4 to 201.9)
Range	0 to 1398	0 to 1764	0 to 3487	0 to 3712
Missing	*2 (3.4%)*	*2 (3.1%)*	*0 (0%)*	*0 (0%)*
**Anti-N Ig**				
negative	26 (45.6%)	26 (41.9%)	25 (39.1%)	19 (33.9%)
positive	31 (54.4%)	36 (58.1%)	39 (60.9%)	37 (66.1%)
Missing	*2 (3.4%)*	*2 (3.1%)*	*0 (0%)*	*0 (0%)*
**Anti-N Ig (COI)**				
Mean (SD)	16.7 (27.2)	33.4 (45.6)	50.2 (63.3)	34.1 (48.5)
Median (IQR)	2.1 (0.1 to 20.1)	7.3 (0.1 to 51.4)	16.8 (0.1 to 86.0)	8.4 (0.1 to 47.5)
Range	0 to 108	0 to 154	0 to 190	0 to 184
Missing	*2 (3.4%)*	*2 (3.1%)*	*0 (0%)*	*0 (0%)*
**Neutralization Assays**				
**Anti-Wildtype neutralizing activity**				
negative	35 (60.3%)	33 (55.0%)	40 (69.0%)	38 (76.0%)
positive	23 (39.7%)	27 (45.0%)	18 (31.0%)	12 (24.0%)
Missing	*1 (1.7%)*	*4 (6.3%)*	*6 (9.4%)*	*6 (10.7%)*
**Anti-Wildtype neutralizing activity (IC_{50})**				
Mean (SD)	164.3 (422.7)	221.3 (692.5)	71.2 (135.0)	33.4 (45.6)
Median (IQR)	39.2 (1.1 to 126.7)	42.5 (1.0 to 113.8)	22.6 (2.4 to 64.2)	14.0 (1.9 to 47.5)

Range	0 to 2550	0 to 4892	0 to 753	0 to 194
Missing	*1 (1.7%)*	*4 (6.3%)*	*6 (9.4%)*	*6 (10.7%)*
**Anti-Delta neutralizing activity**				
negative	50 (86.2%)	51 (85.0%)	53 (91.4%)	49 (98.0%)
positive	8 (13.8%)	9 (15.0%)	5 (8.6%)	1 (2.0%)
Missing	*1 (1.7%)*	*4 (6.3%)*	*6 (9.4%)*	*6 (10.7%)*
**Anti-Delta neutralizing activity (IC_{50})**				
Mean (SD)	41.8 (134.6)	62.9 (241.1)	20.0 (40.5)	10.4 (16.1)
Median (IQR)	5.1 (1.0 to 22.6)	5.1 (1.0 to 25.7)	4.9 (1.0 to 19.8)	2.9 (1.0 to 14.5)
Range	0 to 977	0 to 1813	0 to 211	0 to 83
Missing	*1 (1.7%)*	*4 (6.3%)*	*6 (9.4%)*	*6 (10.7%)*
**Anti-Omicron neutralizing activity**				
negative	57 (98.3%)	56 (98.2%)	48 (98.0%)	41 (100.0%)
positive	1 (1.7%)	1 (1.8%)	1 (2.0%)	0 (0.0%)
Missing	*1 (1.7%)*	*7 (10.9%)*	*15 (23.4%)*	*15 (26.8%)*
**Anti-Omicron neutralizing activity (IC_{50})**				
Mean (SD)	4.6 (20.3)	3.4 (8.3)	4.4 (10.0)	2.4 (3.4)
Median (IQR)	1.0 (0.9 to 1.2)	1.0 (1.0 to 2.1)	1.0 (1.0 to 3.1)	1.0 (1.0 to 2.8)
Range	0 to 152	0 to 56	0 to 64	0 to 17
Missing	*1 (1.7%)*	*7 (10.9%)*	*15 (23.4%)*	*15 (26.8%)*
**ELISpot T Cell Assays**				
**M-specific T cells**				
negative	11 (19.6%)	28 (43.8%)	28 (43.8%)	29 (52.7%)
positive	45 (80.4%)	36 (56.2%)	36 (56.2%)	26 (47.3%)
Missing	*3 (5.1%)*	*0 (0%)*	*0 (0%)*	*1 (1.8%)*
**N-specific T cells**				
negative	15 (26.8%)	28 (43.8%)	28 (43.8%)	27 (49.1%)
positive	41 (73.2%)	36 (56.2%)	36 (56.2%)	28 (50.9%)
Missing	*3 (5.1%)*	*0 (0%)*	*0 (0%)*	*1 (1.8%)*
**S1-specific T cells**				
negative	15 (26.8%)	27 (42.2%)	28 (43.8%)	30 (54.5%)
positive	41 (73.2%)	37 (57.8%)	36 (56.2%)	25 (45.5%)
Missing	*3 (5.1%)*	*0 (0%)*	*0 (0%)*	*1 (1.8%)*
**S2-specific T cells**				
negative	16 (28.6%)	24 (37.5%)	22 (34.4%)	28 (50.9%)
positive	40 (71.4%)	40 (62.5%)	42 (65.6%)	27 (49.1%)
Missing	*3 (5.1%)*	*0 (0%)*	*0 (0%)*	*1 (1.8%)*
**Overall (M, N, S1, or S2 epitope pool-specific) T cells**				
negative	9 (16.1%)	13 (20.3%)	12 (18.8%)	16 (29.1%)
positive	47 (83.9%)	51 (79.7%)	52 (81.2%)	39 (70.9%)
Missing	*3 (5.1%)*	*0 (0%)*	*0 (0%)*	*1 (1.8%)*

Supplementary Table 4. **Sensitivity analysis regarding anti-S IgA and IgG antibody responses, weighted by age group.** Positivity for anti-S IgA, IgG, and either anti-S IgA or IgG (using MFI ratio cutoff values of 6.5 for IgA and 6.0 for IgG), based on the primary analysis (unweighted) and based on a sensitivity analysis applying weighting by age groups based on the age group distribution among all identified cases in the Canton of Zurich with a diagnosed SARS-CoV-2 infection during the study timeframe. Source data are provided as a Source Data file.

	**Assay Positivity**			
	**W2 (N=404)**	**M1 (N=421)**	**M3 (N=418)**	**M6 (N=334)**
**Unweighted Analysis**				
**Anti-S IgA**	82.6%	80.3%	68.9%	70.4%
**Anti-S IgG**	82.4%	83.6%	84.0%	81.4%
**Anti-S IgA or IgG**	84.9%	84.3%	84.7%	84.7%
**Analysis Weighted by Age Strata**				
**Anti-S IgA**	81.3%	78.1%	66.6%	69.6%
**Anti-S IgG**	80.9%	82.4%	82.9%	80.6%
**Anti-S IgA or IgG**	83.4%	83.1%	83.6%	83.8%

Supplementary Table 5. Detailed results for antibody, neutralizing antibody, and T cell decay estimation. Unadjusted and adjusted estimated half-lives (in days) for all immune parameters evaluated in this study based on repeated-measures mixed linear regression models. The adjusted half-life was estimated by adjusting for time from diagnosis to maximum mean fluorescence intensity (MFI) ratio (for anti-S IgA, anti-S IgG, and anti-N IgG), time from diagnosis to maximum half maximal inhibitory concentration (IC_{50}) value (for neutralization assays) or time since diagnosis (for T cell testing), as well as age group, sex and symptom count, using a random intercept for each individual in the study. CI: Confidence Interval, Inf.: Infinity. Source data are provided as a Source Data file.

Measure	Unadjusted Half-Life<br>days (95% CI)	Adjusted Half-Life<br>days (95% CI)
**Overall Study Population (total n=431)**		
Anti-S IgA (Luminex)	70 (65–76)	71 (66–76)
Anti-S IgG (Luminex)	144 (134–155)	145 (135–156)
**Subsample (total n=64)**		
Anti-S IgA (Luminex)	70 (58–88)	71 (59–90)
Anti-S IgG (Luminex)	143 (112–198)	142 (111–197)
Anti-N IgG (Luminex)	86 (76–98)	86 (76–99)
Anti-S Ig (Roche Elecsys)	178 (91–3638)	186 (93–Inf.)
Anti-N Ig (Roche Elecsys)	97 (69–164)	96 (68–160)
Anti-Wildtype SARS-CoV-2	70 (55–94)	70 (55–94)
Anti-Delta SARS-CoV-2	46 (33–76)	46 (33–77)
Anti-Omicron SARS-CoV-2	not estimable	not estimable
Pooled T Cells	163 (84–3318)	161 (83–2810)
M-specific T cells	138 (79–522)	138 (79–524)
N-specific T cells	258 (112–Inf.)	251 (110–Inf.)
S1-specific T cells	136 (80–447)	137 (81–448)
S2-specific T cells	379 (133–Inf.)	382 (134–Inf.)

Supplementary Table 6. **Association between demographic and clinical factors and anti-S IgG antibody responses over time.** Results from univariable (unadjusted) and multivariable (adjusted) repeated-measures mixed linear regression analyses assessing the association of demographic and clinical variables with natural logarithm-transformed anti-S IgG mean fluorescence intensity (MFI) ratios in the overall study population (total n=431). Random intercepts were used for each individual in the study. The multivariable model was adjusted for age group, sex, symptom severity based on symptom count and time from diagnosis to testing. BMI: Body Mass Index, CI: Confidence Interval, ref: Reference. Source data are provided as a Source Data file.

	**Univariable Analysis**		**Multivariable Analysis**	
**Variable**	**Coefficient (95% CI)**	**P-value**	**Coefficient (95% CI)**	**P-value**
**Age group**				
18-39 years	ref		ref	
40-64 years	0.04 (-0.29 to 0.37)	0.81	0.10 (-0.21 to 0.42)	0.53
65+ years	0.70 (0.36 to 1.03)	<0.001	0.78 (0.47 to 1.10)	<0.001
**Sex**				
Female	ref		ref	
Male	0.25 (-0.02 to 0.53)	0.074	0.34 (0.08 to 0.60)	0.011
**Symptom severity (based on symptom count)**				
Asymptomatic	ref		ref	
1-5 symptoms	0.62 (0.24 to 1.00)	0.002	0.66 (0.29 to 1.03)	<0.001
≥6 symptoms	1.15 (0.77 to 1.52)	<0.001	1.28 (0.92 to 1.65)	<0.001
**Hospitalization within first 2 weeks**				
Non-hospitalized	ref		ref	
Hospitalized	1.46 (0.75 to 2.17)	<0.001	0.95 (0.27 to 1.62)	0.006
**Smoking status**				
Non-Smoker	ref		ref	
Ex-Smoker	0.09 (-0.24 to 0.41)	0.61	-0.04 (-0.35 to 0.27)	0.79
Smoker	-0.53 (-0.93 to -0.12)	0.012	-0.50 (-0.88 to -0.12)	0.01
**BMI**				
Per unit increase	0.03 (-0.01 to 0.06)	0.10	0.02 (-0.02 to 0.05)	0.37
**Comorbidities**				
None	ref		ref	
At least one	0.25 (-0.05 to 0.56)	0.10	-0.02 (-0.32 to 0.28)	0.92
**Immune suppression**				
None	ref		ref	
At least one	-0.03 (-0.89 to 0.84)	0.95	-0.28 (-1.07 to 0.52)	0.50

Supplementary Table 7. **Association between demographic and clinical factors and anti-S IgA antibody responses over time.** Results from univariable (unadjusted) and multivariable (adjusted) repeated-measures mixed linear regression analyses assessing the association of demographic and clinical variables with natural logarithm-transformed anti-S IgA mean fluorescence intensity (MFI) ratios in the overall study population (total n=431). Random intercepts were used for each individual in the study. The multivariable model was adjusted for age group, sex, symptom severity based on symptom count and time from diagnosis to testing. BMI: Body Mass Index, CI: Confidence Interval, ref: Reference. Source data are provided as a Source Data file.

	Univariable Analysis		Multivariable Analysis	
**Variable**	**Coefficient (95% CI)**	**P-value**	**Coefficient (95% CI)**	**P-value**
**Age group**				
18-39 years	ref		ref	
40-64 years	0.30 (-0.07 to 0.66)	0.11	0.34 (-0.02 to 0.69)	0.063
65+ years	0.82 (0.45 to 1.19)	<0.001	0.82 (0.46 to 1.17)	<0.001
**Sex**				
Female	ref		ref	
Male	0.44 (0.14 to 0.74)	0.005	0.52 (0.22 to 0.81)	<0.001
**Symptom severity (based on symptom count)**				
Asymptomatic	ref		ref	
1-5 symptoms	0.39 (-0.04 to 0.82)	0.078	0.46 (0.04 to 0.87)	0.031
≥6 symptoms	0.86 (0.44 to 1.28)	<0.001	1.05 (0.64 to 1.46)	<0.001
**Hospitalization within first 2 weeks**				
Non-hospitalized	ref		ref	
Hospitalized	1.36 (0.57 to 2.15)	<0.001	0.87 (0.10 to 1.63)	0.026
**Smoking status**				
Non-Smoker	ref		ref	
Ex-Smoker	0.13 (-0.23 to 0.49)	0.48	-0.06 (-0.41 to 0.30)	0.75
Smoker	-0.47 (-0.92 to -0.02)	0.042	-0.43 (-0.85 to 0.00)	0.051
**BMI**				
Per unit increase	0.04 (0.00 to 0.08)	0.041	0.01 (-0.02 to 0.05)	0.49
**Comorbidities**				
None	ref		ref	
At least one	0.29 (-0.05 to 0.63)	0.093	-0.02 (-0.36 to 0.32)	0.91
**Immune suppression**				
None	ref		ref	
At least one	0.07 (-0.87 to 1.02)	0.88	-0.15 (-1.05 to 0.75)	0.75

Supplementary Table 8. **Association between demographic and clinical factors and pooled T cell responses over time.** Results from univariable (unadjusted) and multivariable (adjusted) repeated-measures mixed linear regression analyses assessing the association of demographic and clinical variables with natural logarithm-transformed pooled T cell responses (summed M, N, S1, and S2 spot-forming units (SFU) per 1e6 peripheral blood mononuclear cells (PBMCs)) in the subsample selected for detailed analyses (total n=64). Random intercepts were used for each individual in the study. The multivariable model was adjusted for age group, sex, symptom severity based on symptom count and time from diagnosis to testing. BMI: Body Mass Index, CI: Confidence Interval, ref: Reference. Source data are provided as a Source Data file.

	Univariable Analysis		Multivariable Analysis	
**Variable**	**Coefficient (95% CI)**	**P-value**	**Coefficient (95% CI)**	**P-value**
**Age group**				
18-39 years	ref		ref	
40-64 years	0.27 (-0.63 to 1.18)	0.55	0.23 (-0.57 to 1.03)	0.57
65+ years	1.45 (0.50 to 2.41)	0.003	1.58 (0.70 to 2.46)	<0.001
**Sex**				
Female	ref		ref	
Male	0.04 (-0.79 to 0.87)	0.92	0.11 (-0.57 to 0.79)	0.75
**Symptom severity (based on symptom count)**				
Asymptomatic	ref		ref	
1-5 symptoms	0.17 (-0.79 to 1.13)	0.72	0.73 (-0.19 to 1.64)	0.12
≥6 symptoms	1.45 (0.56 to 2.34)	0.002	1.76 (0.94 to 2.58)	<0.001
**Hospitalization within first 2 weeks**				
Non-hospitalized	ref		ref	
Hospitalized	1.24 (0.14 to 2.34)	0.028	-0.08 (-1.18 to 1.03)	0.89
**Smoking status**				
Non-Smoker	ref		ref	
Ex-Smoker	1.23 (0.33 to 2.12)	0.008	0.98 (0.20 to 1.77)	0.015
Smoker	0.49 (-0.51 to 1.50)	0.33	0.43 (-0.43 to 1.29)	0.32
**BMI**				
Per unit increase	0.00 (-0.09 to 0.09)	0.96	-0.01 (-0.09 to 0.07)	0.88
**Comorbidities**				
None	ref		ref	
At least one	0.99 (0.11 to 1.88)	0.029	0.42 (-0.41 to 1.25)	0.32
**Immune suppression**				
None	ref		ref	
At least one	-0.57 (-3.73 to 2.58)	0.72	-0.70 (-3.40 to 2.01)	0.61

Supplementary Table 9. **Association between demographic and clinical factors and anti-S IgG antibody positivity at two weeks and six months.** Results from multivariable (adjusted) mixed logistic regression analyses assessing the association of demographic and clinical variables with anti-S IgG antibody positivity (defined as a mean fluorescence intensity (MFI) ratio above 6.0) in the overall study population (total n=431) at two weeks and six months after diagnosis of SARS-CoV-2 infection. Models were adjusted for age group, sex, symptom severity based on symptom count and time from diagnosis to testing. Odds ratios for hospitalization could not be meaningfully estimated since all hospitalized participants were tested antibody positive. BMI: Body Mass Index, CI: Confidence Interval, OR: Odds Ratio, n.e.: Not estimated, ref: Reference. Source data are provided as a Source Data file.

	**Anti-S IgG Positivity at 2 Weeks**		**Anti-S IgG Positivity at 6 Months**	
**Variable**	**OR (95% CI)**	**P-value**	**OR (95% CI)**	**P-value**
**Age group**				
18-39 years	ref		ref	
40-64 years	0.85 (0.45 to 1.61)	0.62	0.89 (0.47 to 1.69)	0.72
65+ years	1.87 (0.91 to 3.95)	0.093	1.56 (0.70 to 3.68)	0.29
**Sex**				
Female	ref		ref	
Male	1.98 (1.15 to 3.48)	0.015	1.62 (0.91 to 2.95)	0.11
**Symptom severity (based on symptom count)**				
Asymptomatic	ref		ref	
1-5 symptoms	1.87 (0.97 to 3.62)	0.062	1.35 (0.63 to 2.83)	0.43
≥6 symptoms	6.25 (2.94 to 13.72)	<0.001	4.23 (1.88 to 9.67)	<0.001
**Hospitalization within first 2 weeks**				
Non-hospitalized	ref		ref	
Hospitalized	n.e.	n.e.	n.e.	n.e.
**Smoking status**				
Non-Smoker	ref		ref	
Ex-Smoker	0.62 (0.31 to 1.26)	0.18	0.76 (0.37 to 1.65)	0.48
Smoker	0.24 (0.12 to 0.51)	<0.001	0.34 (0.16 to 0.74)	0.006
**BMI**				
Per unit increase	0.99 (0.92 to 1.07)	0.72	0.97 (0.90 to 1.06)	0.51
**Comorbidities**				
None	ref		ref	
At least one	0.82 (0.43 to 1.59)	0.56	1.15 (0.57 to 2.45)	0.70
**Immune suppression**				
None	ref		ref	
At least one	0.66 (0.15 to 4.72)	0.63	0.57 (0.12 to 4.17)	0.52

Supplementary Table 10. **Association between demographic and clinical factors and overall T cell positivity at two weeks and six months.** Results from multivariable (adjusted) mixed logistic regression analyses assessing the association of demographic and clinical variables with overall T cell positivity (defined as as a spot-forming unit (SFU) value of greater than 0 to one or more peptide pools (M, N, S1, or S2)). in the subsample selected for detailed analyses (total n=64) at two weeks and six months after diagnosis of SARS-CoV-2 infection. Models were adjusted for age group, sex, symptom severity based on symptom count and time from diagnosis to testing. Odds ratios for immune suppression could not be meaningfully estimated. BMI: Body Mass Index, CI: Confidence Interval, OR: Odds Ratio, n.e.: Not estimated, ref: Reference. Source data are provided as a Source Data file.

		**Overall T Cell Positivity at 2 Weeks**		**Overall T Cell Positivity at 6 Months**	
**Variable**		**OR (95% CI)**	**P-value**	**OR (95% CI)**	**P-value**
**Age group**					
	18-39 years	ref		ref	
	40-64 years	0.34 (0.03 to 2.58)	0.31	0.95 (0.22 to 4.12)	0.95
	65+ years	2.08 (0.16 to 28.91)	0.57	0.72 (0.13 to 4.18)	0.71
**Sex**					
	Female	ref		ref	
	Male	2.80 (0.54 to 18.86)	0.24	0.73 (0.19 to 2.77)	0.64
**Symptom severity (based on symptom count)**					
	Asymptomatic	ref		ref	
	1-5 symptoms	5.92 (0.81 to 66.09)	0.10	1.30 (0.25 to 6.78)	0.75
	≥6 symptoms	11.08 (1.17 to 192.65)	0.057	4.22 (0.88 to 23.09)	0.079
**Hospitalization within first 2 weeks**					
	Non-hospitalized	ref		ref	
	Hospitalized	n.e.	n.e.	5.13 (0.39 to 167.54)	0.27
**Smoking status**					
	Non-Smoker	ref		ref	
	Ex-Smoker	0.73 (0.05 to 9.14)	0.80	27.89 (3.15 to 738.56)	0.011
	Smoker	0.03 (0.00 to 0.42)	0.029	3.00 (0.44 to 28.21)	0.28
**BMI**					
	Per unit increase	0.95 (0.80 to 1.14)	0.60	0.91 (0.77 to 1.06)	0.23
**Comorbidities**					
	None	ref		ref	
	At least one	2.82 (0.40 to 28.35)	0.32	3.84 (0.59 to 38.59)	0.19
**Immune suppression**					
	None	ref		ref	
	At least one	n.e.	n.e.	n.e.	n.e.

Supplementary Table 11. **Reference table for converting anti-S IgG MFI ratios to U/ml.** Equivalence values between the Luminex-based mean fluorescence intensity (MFI) ratios and U/ml based on the Roche Elecsys Anti-SARS-CoV-2 S Ig immunoassay, derived from validation studies (Supplementary Fig. 1f). Limit of detection cut-off for IgG MFI ratios for Luminex assay is 6.0.

**Luminex Anti-S IgG (MFI Ratio)**	**Roche Elecsys Anti-S Ig (U/ml)**
1	1
4	6
6	12
10	30
25	170
50	656
100	2585
150	5795
200	10289
