# Supplementary material for: Heterogenous humoral and cellular immune responses with distinct trajectories post-SARS-CoV-2 infection in a population-based cohort
Source: Nat Commun. 2022 Aug 18;13:4855. doi: 10.1038/s41467-022-32573-w (PMC9386650; doi:10.1038/s41467-022-32573-w)
Supplement: Supplementary file 3 — Description of Additional Supplementary Files [file 41467_2022_32573_MOESM3_ESM.pdf]

Supplementary Code 1: R code allowing to reproduce the results of the study
